# Characterization of the disruption of neural control strategies for dynamic fingertip forces from attractor reconstruction

**DOI:** 10.1371/journal.pone.0172025

**Published:** 2017-02-13

**Authors:** Lorenzo Peppoloni, Emily L. Lawrence, Emanuele Ruffaldi, Francisco J. Valero-Cuevas

**Affiliations:** 1 PERCRO Laboratory, TeCIP Institute, Scuola Superiore Sant’Anna, via Alamanni 13b, 56010 Ghezzano, San Giuliano Terme, Pisa, Italy; 2 Brain-Body Dynamics Laboratory, Department of Biomedical Engineering, University of Southern California, 3710 McClintock Ave., Los Angeles, CA, 90089, United States of America; 3 Brain-Body Dynamics Laboratory, Department of Biomedical Engineering & Division of Biokinesiology and Physical Therapy, University of Southern California, 3710 McClintock Ave., Los Angeles, CA, 90089, United States of America; University of Chicago, UNITED STATES

## Abstract

The Strength-Dexterity (SD) test measures the ability of the pulps of the thumb and index finger to compress a compliant and slender spring prone to buckling at low forces (<3N). We know that factors such as aging and neurodegenerative conditions bring deteriorating physiological changes (e.g., at the level of motor cortex, cerebellum, and basal ganglia), which lead to an overall loss of dexterous ability. However, little is known about how these changes reflect upon the dynamics of the underlying biological system. The spring-hand system exhibits nonlinear dynamical behavior and here we characterize the dynamical behavior of the phase portraits using attractor reconstruction. Thirty participants performed the SD test: 10 young adults, 10 older adults, and 10 older adults with Parkinson’s disease (PD). We used delayed embedding of the applied force to reconstruct its attractor. We characterized the distribution of points of the phase portraits by their density (number of distant points and interquartile range) and geometric features (trajectory length and size). We find phase portraits from older adults exhibit more distant points (*p* = 0.028) than young adults and participants with PD have larger interquartile ranges (*p* = 0.001), trajectory lengths (*p* = 0.005), and size (*p* = 0.003) than their healthy counterparts. The increased size of the phase portraits with healthy aging suggests a change in the dynamical properties of the system, which may represent a weakening of the neural control strategy. In contrast, the distortion of the attractor in PD suggests a fundamental change in the underlying biological system, and disruption of the neural control strategy. This ability to detect differences in the biological mechanisms of dexterity in healthy and pathological aging provides a simple means to assess their disruption in neurodegenerative conditions and justifies further studies to understand the link with the physiological changes.

## Introduction

**Fig 1:**
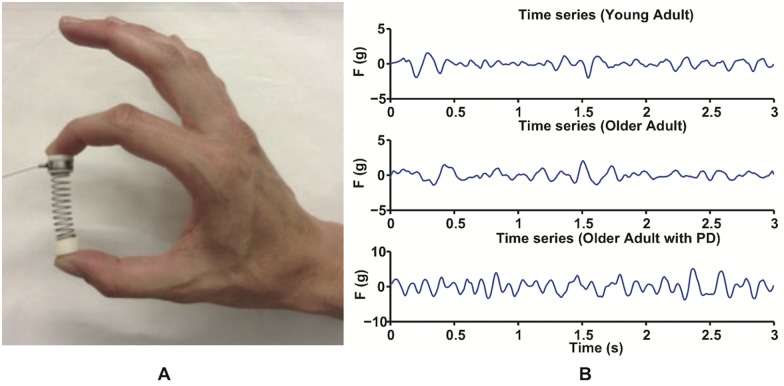
The SD test. The SD test (A) consists of compressing a compliant, slender spring prone to buckling, and once the maximal level of compression has been reached, it is sustained for at least 3s. The pulps of the thumb and index finger press the end caps of the spring and a miniature load cell is positioned under the index and thumb fingers. (B). Three filtered force traces for a young adult (top), an older adult (center), and an older adult with PD (bottom) are shown.

The ability to dynamically regulate the direction of fingertip force vectors of low magnitudes (e.g., dexterity), is essential for everyday activities and greatly influences quality of life [1, 2]. Sudden or gradual losses of dexterity and/or impaired neural control of manipulation can lead to difficulties in performing activities of daily living (ADLs) [3]. For example, aging and some clinical conditions (i.e., Parkinson’s disease, PD) result in progressive losses of dexterity [4, 5]. Thus, assessing and quantifying one’s ability to dynamically regulate fingertip forces becomes particularly important to study healthy aging and clinical conditions. As a result, numerous tests of fingertip force production are used to assess hand function [6, 7]. In particular, the Strength-Dexterity (SD) paradigm quantifies the ability to use fingertip forces to compress and hold a slender spring prone to buckling [1] (Fig 1A and 1B). The spring becomes increasingly unstable when compressed, and the average maximal level of sustained compression a person can achieve has been used as a quantitative metric of the sensorimotor ability for dexterous manipulation [1, 2]—and even for dexterous foot-ground interactions when compressing a larger spring with the leg [8]. This discrete metric has successfully quantified the effects of development, aging [4, 8, 9] and clinical conditions [5, 8, 10, 11] on manipulation ability. Although prior work has used statistical and spectral analyses of the average forces at the edge of instability, a formal dynamical analysis of the time-varying fingertip forces that achieve the maximal sustained compression should be more informative of the biological dynamical system underlying dexterous manipulation, its healthy properties and as a consequence how its behavior is affected by diseased states. In particular, one study has concluded that—when at the edge of instability—the combined system of the fingers, spring and neuromuscular system behaves as a nonlinear dynamical system in the vicinity of a subcritical pitchfork bifurcation [12]. For this reason, a nonlinear analysis of the time history of forces may be able to reveal the properties of the dynamical “attractor”, which standard linear techniques cannot do [13]. Unfortunately, the few seconds that subjects can reliably remain at the edge of instability produce short time series that are not well-suited to many nonlinear dynamical measures (i.e, maximal Lyapunov Exponent, Correlation Dimension) that require long time series [14, 15].

Attractor reconstruction, however, has been successfully used as a tool to characterize the variability and stability of dynamic biological systems [16]. For example, attractor reconstruction can characterize the level of anesthesia [17] and classify epileptic seizures [18] when applied to electroencephalographic signals, assess heart function when applied to electrocardiograms [19], and characterize neuromuscular function when applied to the variability of static forces [20–23]. This technique is informative since the reconstructed attractor is presumed to be topologically equivalent to the original non-observable attractor [24]. If the reconstructed phase space (the space in which all possible states of a system are represented, with each possible state corresponding to one unique point in the phase space) shows systematic, condition-related changes in its topology, then the underlying dynamics of the system are also changing [25].

Here we focus on attractor reconstruction as a geometric characterization of the effects of age and PD on the ability to stabilize an unstable object with the fingertips. We use the time histories of fingertip forces to reconstruct the attractors that characterize the hand-spring-neural control system of manipulation at the edge of instability—and to quantify differences in the temporal and geometric structure of the attractors among young adults, older adults with PD, and age-matched healthy older adults. The quantified differences represent the consequence of the changes at the physiological level, however, further investigation will be necessary to understand the link between our results and the physiology.

## Materials and methods

### Definitions and motivation

The nonlinear analysis detailed in this paper is based on the theory of nonlinear dynamical systems, where the time evolution of a system is defined in a phase space. In a nonlinear system that is purely deterministic, all its future states are fixed once the present state is fixed. But it can be chaotic if small differences in initial conditions yield widely diverging outcomes, rendering long-term prediction impossible. Thus, generally speaking, nonlinear systems may exhibit deterministic chaos. To study such systems, we can usually assume that the stochastic component is small and does not change the fundamental nonlinear properties of the system. We can then define a vector space, namely a *state space* or *phase space* for the system. Every point in the state space specifies a state of the system and vice versa. This property allows us to study the dynamics of the system through the study of the points it visits in state space. Note that, except for dynamical models with defined mathematical equations of motion, there is usually no unique choice for the phase space of experimental systems. In the case of nondeterministic systems, we can still consider the concept of state space, but usually by only taking into account a set of states and transition rules between them [24]. For deterministic systems, we can usually find their finite *m*-dimensional vector space, where the state is defined by a vector *x* ∈ ℜ^*m*^. If the system is discrete, its dynamics are described by an *m*-dimensional map *x*_*n*+1_ = *F*(*x*_*n*_). If the system is continuous, its dynamics are defined by a set of *m* first-order differential equations, $\frac{d}{dt}x\left(t\right)=f\left(x\left(t\right)\right)$.

A sequence of points that represent a solution to the above equation given some initial conditions is called a *trajectory* of the dynamical system. A geometric representation of the trajectories of the system in the phase space is called a *phase portrait*. For a system with bounded solutions and dissipative tendencies (meaning that on average the volume of the phase space containing the initial conditions tends to contract with the evolution of the system state), a set of initial conditions will evolve towards (i.e., be attracted to) a certain subset of the phase space. This subset is defined an *attractor* for the system, and it is invariant under the system’s dynamical evolution. Examples of attractors are fixed points and limit cycles [24]. In the case of deterministically chaotic systems, attractors may exhibit very complicated geometrical structures, for this reason they are usually called *strange attractors* [26]. The properties of the attractor, such as type, shape, location, and size, are dependent upon the values of the parameters of the dynamic system, and for this reason the investigation of the phase portraits (i.e what attractor exists and what are its properties) can shed light on the nature of underlying dynamical system [27]—and in this case the biological controller that produces the stabilization of the spring.

### Participant demographics

We re-analyzed the fingertip forces that ten young adults (6F, 4M, mean±SD, 24.1±1.2 yrs), ten healthy older adults (5F, 5M, 65.2±6.7 yrs), and ten older adults with PD (6F, 4M, 68.1±8.9 yrs) used to perform the SD test, as reported in prior studies [5, 8]. All participants gave their written, informed consent prior to participation and the Institutional Review Board at the University of Southern California (Los Angeles, CA, USA) approved the study protocol. The individual in this manuscript has given written informed consent (as outlined in PLOS consent form) to publish these case details.

### Data collection

The published SD test consists of a 3.96 cm spring outfitted with a miniature force sensor positioned under the index finger (Measurement Specialties, Hampton, VA). Subjects were asked to compress the spring with only their thumb and index finger to the point of maximal instability they can sustain (i.e., beyond which they feel it would slip out of their fingertips) and maintain a constant level of compression [9]. Data acquisition hardware (National Instruments, Austin, TX) sampled the conditioned signal of the sensors at 2000 Hz and we used custom MATLAB (v2015b, Mathworks, Natick, MA) software to process and analyze the data. We used the same hold phases, defined as the periods of maximal sustained compression with the fingers (10 for each participant) reported previously [5, 8]. In our prior work, we analyzed the three hold phases with the highest mean compression forces held stable for at least three seconds. However, for this nonlinear analysis, instead of calculating the average maximal sustained force, its dispersion, or frequency content, we used the force time histories during all ten hold phases. The force traces from index and thumb finger were averaged, downsampled to 400 Hz and bandpass-filtered (Butterworth, 3–30 Hz) to focus our analysis on the force variability related to fast corrections and reflexive actions, but which unavoidably also includes physiological and pathological tremor [5, 22, 23, 28].

### Attractor reconstruction

Real-world dynamical systems are generally too complex to directly observe the underlying attractors. Typically, not all the variables involved are observable and, moreover, sampling and quantization effects represent a breach of the differentiability whose validity is also substantially weakened in the presence of unavoidable experimental, measurement or physiological noise. For these reasons, methods have been developed to reconstruct a mapping function between the one-dimensional observed variable (the time series of force) and its attractor (if it exists). The goal is to obtain a phase portrait which preserves the topological and dynamical properties of the original system [29] while revealing some properties of the underlying attractor. One such tool for attractor reconstruction is the delayed embedding theorem [29], stating that the vector sequence,

$$\begin{array}{c} Y\left(i\right)=\left(y_{i},y_{i+\tau },y_{i+2\tau },\ldots ,y_{i+(m-1)\tau }\right) \end{array}$$ (1)

provides a reconstructed attractor with the same properties of the original system; where *y*_*i*_ is the value of the time series at time *i*, tau (*τ*) is the embedding delay, and *m* is the embedding dimension. The underlying idea is that the variables in a deterministic dynamical system are generically connected, influencing one another. Every subsequent point of a given measurement *y*_*i*_ is the result of a combination of the influences from all other variables of the system up to a certain value of lag, after which the memory of the previous state of the system is lost. For this reason, it can be treated as a substitute second system variable (or heuristic state variable), which carries information about the influence of all other variables during the time interval *τ*. By the same reasoning, all the other substitute delayed coordinates can be introduced by obtaining the *m*-dimensional phase portrait (in the *m*-dimensional heuristic state space), provided an appropriately large enough *m*. It is crucial to state that the information carried by the heuristic variables is identical to that carried by the original (but hidden) system variables with the exception that properties associated with the system’s dynamics have no particular physical meaning [30].

**Fig 2:**
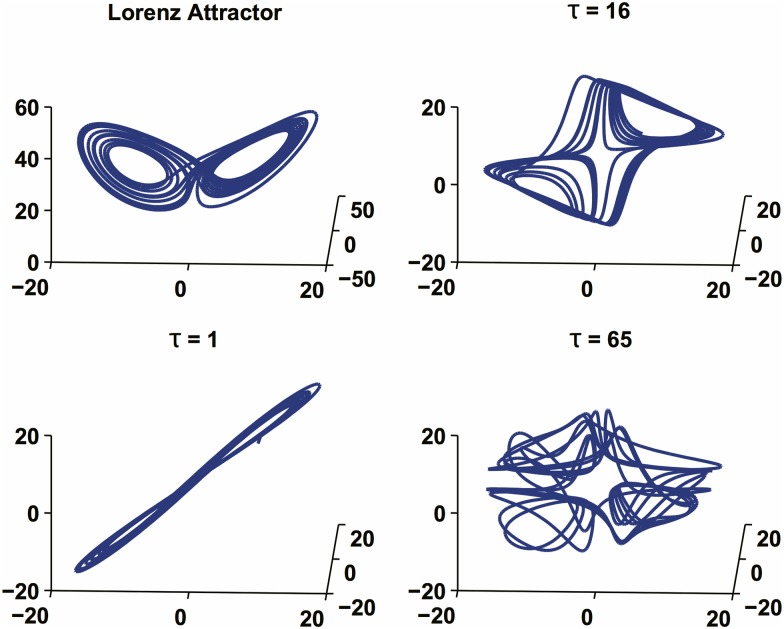
Effects of the embedding delay on the reconstructed attractor. The exact phase portrait of the dual attractor (top left) and a correct reconstruction (top right) are shown. When *τ* is chosen too small (bottom left) the reconstructed attractor appears compressed without well-evolved folding regions. When chosen too large (bottom right), the resulting attractor shows trajectories folding and wrapping around very frequently, with the resulting fragmentation of the dual attractor and the introduction of a seemingly stochastic nature.

We emphasize that the embedding parameters *τ* and *m* must be properly chosen. The embedding delay *τ* must be large enough so that the information gained from measuring the value of *y*_*i*_ + *y*_(*i* + *τ*)_ is significantly different from the information already known from the value of *y*_*i*_. This will allow the proper “unfolding” of the attractor in the phase space. Conversely, *τ* should not be larger than the typical time interval in which the system loses memory of its prior state. Fig 2 shows an example of the influence of the choice of *τ* in the reconstruction of the well known Lorenz Attractor. When *τ* is chosen properly (top right), the reconstruction “unfolds” correctly off the main diagonal. If *τ* is too small (bottom left), the *m* coordinates of each attractor point are strongly correlated and the embedded dynamics lie in the proximity of the main diagonal of the phase space. Conversely, if *τ* is too large (bottom right), the reconstructed phase space consists of uncorrelated points, resulting in a randomly shaped attractor.

**Fig 3:**
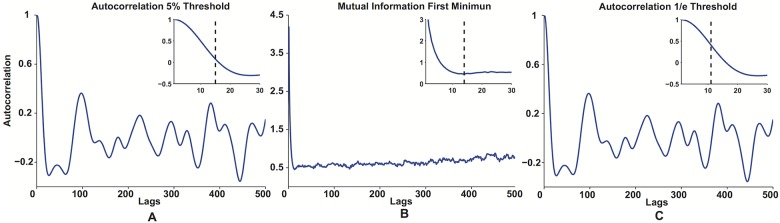
Examples of the choice of the embedding delay *τ*. The autocorrelation function (A, B) and mutual information (C) up to a lag of 500 are shown for a force trace from one of the collected hold phases (older adult). The insets show the zoom of the point where the 5% threshold is crossed by the autocorrelation (A), where the first minimum of the mutual information is (B) and where the 1/*e* threshold is crossed by the autocorrelation (C).

In order to determine the appropriate *τ*, statistics that measure the independence of separated points in the time series are often employed. For example, the first zero crossing threshold of the autocorrelation function [24] yields the smallest value that maximizes the linear independence of the coordinates of the embedding vector. Other thresholds have also been proposed for the autocorrelation, such as 1/*e* or its 5% value [17]. Another approach is to utilize the first minimum of the mutual information function [19], since it adds the largest amount of known data from the previous point of the time series, without completely losing the correlation between the points themselves. Fig 3 shows examples of different thresholds for a force trace from a healthy older adult. To find a conservative estimate for *τ*, we used all three methods to calculate the embedding delay for all ninety hold phases, and then created histograms of the values. The mode of the distributions, thus the value that appears most often for the data in all the three methods, was chosen as the embedding delay.

The embedding dimension, *m*, should be the lowest dimension that allows the dynamics of the attractor to properly topologically unfold. When the embedding dimension is too small, there is a loss of geometrical information much like observing the 2-D shadow of a 3-D object rather than the object itself. There is a “flattening” of the shape and points that are far from each other in the 3-D object are projected closer to each other in the lower dimension. This geometrical property can be exploited to compute the proper embedding dimension using the false nearest neighbors method [31].

In this method, the data are first embedded in a chosen dimension m*, each point’s near neighbors are computed. The embedding dimension is increased (m* + 1) and near-neighbor are re-calculated. If some neighbor in m* dimensions is false, that is, it is no longer a neighbor in m* + 1 dimensions, this is an indication that the dynamics were not properly unfolded. Formally, for a given *m*, for every point *p*_*i*_ in the *m*-dimensional space, a near neighbor *p*_*j*_ is taken (*p*_*j*_ : ∥*p*_*i*_ − *p*_*j*_∥_2_ < *ε*) and the normalized distance in the *m*+1-dimensional space is computed as:

$$\begin{array}{c} R_{i}=\frac{|y_{i+m\tau }-y_{j+m\tau }|}{∥p_{i}-p_{j}{∥}_{2}}. \end{array}$$ (2)

If the distance *R*_*i*_ is smaller than a chosen threshold *R*_*th*_, the points have a false nearest neighbor. When the embedding dimension *m* is chosen high enough, the ratio of the false neighbors is zero or sufficiently small. Usually the threshold distance is chosen such that 0 < *R*_*th*_ < 10 and 0 < *ε* < 0.1*σ*, where *σ* is the standard deviation of the time series.

**Fig 4:**
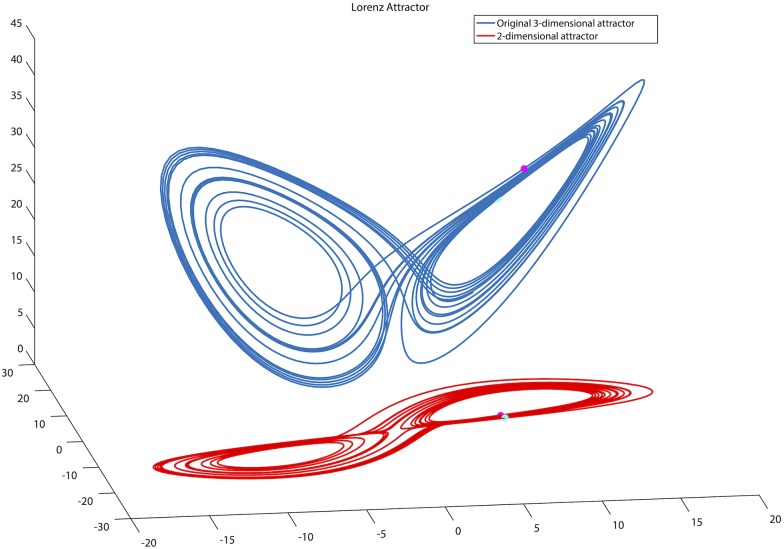
Effects of the embedding dimension on the reconstructed attractor. The exact phase portrait of the dual attractor (blue), with *m* = 3 and a reconstruction with *m* = 2 (red) are shown. When *m* is too small points in the phase space which are far apart in the correct dimension may be closer in a smaller embedding dimension, thus the reconstructed attractor shows partially developed dynamics.

Fig 4 shows an example of the influence of the choice of *m* in the reconstruction of the Lorenz Attractor. In blue the original 3-D attractor is shown, together with the reconstructed attractor with *m* = 2 in red. Two points (cyan and magenta) which are far apart in the original attractor, may lay closer to each other in a lower dimensional space, due to the effects of projection.

Note that this approach allows the original and the reconstructed attractor to share the same topology and the same geometrical form (Fig 2), thus justifying the investigation of the spatial properties of the reconstructed attractor.

### Spatial features of the phase portrait

Once we reconstructed the attractors by creating the phase portraits with the appropriate embedding dimension, *m*_0_, we used several geometric features to characterize their spatial properties [17] (i.e., a means to quantify their topology and geometrical form). Each feature provides a quantitative index of the geometric and distribution properties of the reconstructed attractors that speaks to characterizing information of density, perimeter, area, and volume, or their combination.

The first feature we used is the *Length of the Phase Trajectory* (TL) defined as,

$$\begin{array}{c} TL=\sum_{i=1}^{N}∥Y_{i+1}-Y_{i}∥ \end{array}$$ (3)

where Y is the reconstructed phase portrait and N is the number of points that the time series contains (see Eq 1) [17]. With this feature the distance between every consecutive (*m* − 1)*τ*-dimensional point is considered. TL is an indirect measure of the level of stochasticity of the state space. In fact, as a signal becomes more chaotic, two initially close points in the state space move further from each other and consequently have a longer TL.

A second group of features was chosen to measure the spatial distribution of the points in the attractor and, in particular, to quantify the spatial dispersion from the point in the (*m* − 1)*τ*-dimensional space that is the inferred centroid of the attractor. From a control point of view, trajectories in the phase space characterized by points far from the attractor centroid can be the symptom of a weaker dynamical control action, which is less efficient at bringing the system state toward the attractor [32]

The first spatial distribution feature we used is the *Number of Distant Points* (DP) [17]. DP is computed by counting the number of points whose distance is higher than three standard deviations (3*σ*) from the attractor centroid, according to a chosen metric. Taking into account the typical ellipsoid shape characterizing the reconstructed attractors, then the Mahalanobis distance defined as,

$$\begin{array}{c} d_{M}=\sqrt{{\left(x-\mu \right)}^{T}\Sigma^{-1}\left(x-\mu \right)} \end{array}$$ (4)

can be chosen as a distance metric because it takes into account the dispersion of points and correlation between variables, being Σ and *μ* the signal variance and mean, respectively.

The second spatial distribution feature we considered is the *Interquartile Range of the Euclidean Distance from the Centroid* (IQR). In general, the interquartile range measures the statistical dispersion of the distribution of a set of points. In particular, it defines the difference between the 25th and 75th percentile of the distribution of points. Thus it describes the middle 50% of observations. We applied the interquartile range to the distribution of the Euclidean distance of the points belonging to the phase space trajectories from the trajectory centroid. If the interquartile range of the distances is large, it means that the middle 50% of observations are spaced wide apart. When computing IQR for the distance of phase portrait points from the centroid, it provides a measurement of how scattered the points are. It is to be noted that while the IQR captures the spread of the distribution of the 50% of the points of the phase portrait, while the DP captures the number of samples further than 3*σ* from the distribution mean.

Finally, to assess the overall geometry of the reconstructed attractor, we computed its minimum convex hull and we used the *Sum of the Length of the Edges of the convex hull* (SE) and its *Volume* (V) as its representative features [17]. The former is an index of the perimeter or the area of the attractor, while the latter quantifies the spatial spread of the points forming the phase portrait. We note that one limitation of comparing the features of the convex hulls (CH) is that they must be in the same dimension. Convex hulls are a common way to “generalize” the shape of an attractor and extract useful information, which can be interpreted more easily and used for comparison [33].

**Fig 5:**
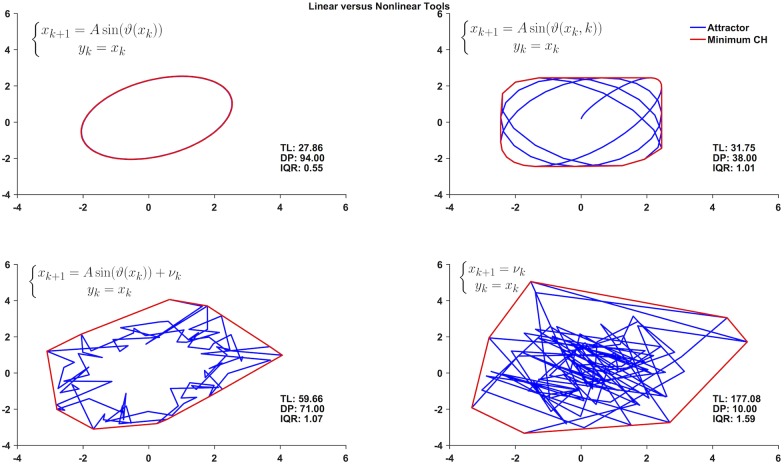
Linear and nonlinear measures of variability. Reconstructed attractors (in blue) in a 2-D embedding space obtained from four time series from three different nonlinear maps. Despite generating time series with the same mean (*μ* = 0.3), standard deviation (*σ* = 1.6) and RMS (*RMS* = 1.6), distinct differences can be highlighted through the spatial characterization of the reconstructed attractor. The shown convex hulls (CH) in red and values for length of the phase trajectory (TL), number of distant points (DP) and interquartile range of the Euclidean distance from the centroid (IQR) reflect the differences in the system dynamics. For example the more chaotic the signal is, the more the reconstructed phase portrait shows stochastic traits with an associated higher level of TL. [17].

Fig 5 illustrates how the proposed nonlinear spatial characterization of the phase space is more informative of the underlying dynamics, compared to traditional linear measures of variability. To demonstrate, we used three nonlinear maps to generate four time series, and to compute the respective convex hull (in red), trajectory length (TL) and the number of distant points (DP) associated with their reconstructed attractors (in blue). The four time series were all characterized by the same standard deviation (*σ* = 1.6), root mean square (RMS) (*RMS* = 1.6) and mean values (*μ* = 0.3), making it impossible to distinguish them with those linear measures. However, the nonlinear spatial characterization was sensitive to the differences in their dynamics reflecting—for example—an increasing level of stochasticity (i.e. increasing values of length of the phase trajectory, TL).

### Ellipsoid fitting

In general, when we consider the phase portraits of biological signals, it can be seen that the points exhibit elliptical shapes [34], where the central area of the ellipse is dense of points, while the peripheral area results more blurred with the possible presence of outliers [17]. The convex hull, being by definition a set of points containing all the fitted points, is sensitive to the presence of outliers and in general to distributions of points with long tails, whose presence leads to a bigger polyhedron. In order to gain a better understanding of the differences among the shapes of the phase portraits for each population, we fit a 3-D ellipsoid to the set of points belonging to each phase space trajectory. For the analysis of the point distribution, an ellipsoid can be interpreted as the equidensity contours of an *m*-dimensional multivariate normal distribution (MVN) centered on the centroid of the points. The eigenvectors of the covariance matrix of the points define the orientation of the ellipsoid principal axes, which are also the directions of the principal components of the data, while the eigenvalues define the squared relative lengths of the principal axes and the proportion of variance explained by that component. For this reason ellipsoid fitting is perfectly equivalent to performing principal components analysis, but we prefer this geometrical interpretation since it is more directly related to the spatial properties of the points distribution. Compared to the convex hull, the ellipsoid fitting technique is robust to the presence of outliers and long tails of points and is more informative about the direction and the level of spread of the data. We compared the lengths of the principal axes of the ellipsoids for the three populations.

We next computed the approximated Sphericity (Ψ) of the ellipsoids for the medians of the axes to assess the differences in the ellipsoid axes among groups. Sphericity measures how spherical an object, higher values indicate a more spherical shape, conversely lower values indicate a less spherical shape. When computed for ellipsoidal objects Ψ is calculated as,

$$\begin{array}{c} \Psi =\frac{\pi^{\frac{1}{3}}{\left(6V\right)}^{\frac{2}{3}}}{A} \end{array}$$ (5)

where *V* is the volume and *A* is the area of the object. In the case of an ellipsoid $V=\frac{4}{3}\pi a_{1}a_{2}a_{3}$ and in the case of a scalene ellipsoid the area is approximated as $A=4\pi {\left(\frac{a_{1}^{p}a_{2}^{p}+a_{1}^{p}a_{3}^{p}+a_{2}^{p}a_{3}^{p}}{3}\right)}^{\frac{1}{p}}$ where *p* = 1.6 and *a*_1_, *a*_2_ and *a*_3_ are the semi-principal axes.

The spatial and geometric characterization of the phase space can be linked to the efficacy and the efficiency of the stabilizing neural control action during the SD test. To understand the relationship between the stabilizing action and the topology of the phase portrait, it can be useful to utilize as an example the task of stabilizing a buckling column. This simple system has three fixed points, the saddle at the origin of the phase space, which is unstable and two stable fixed points, which represent the two possible buckled states. Under the action of an external controller, the column will buckle towards one of the two stable states. What we see when we reconstruct the phase portrait is the “equivalent” stable attractor in which the system has been stabilized during that particular trial. Under a control action which is perfect, the column, and in the same way the spring of the SD test, will reach the stable condition. In the case in which the controller is not able to bring the system state to the stable fixed point, the state trajectories will reach a neighborhood of the fixed point. A reduced control action results in a larger neighborhood. Moreover, the dimension of the phase portrait in the phase space will give a quantification of the efficiency of the controller in bringing the state towards the stable fixed point. We must add that if we compare the same physiological system, in different states (e.g., healthy versus affected by a neurological condition) it is not possible to infer if the differences in the phase portrait are due to a different control action or a different, less controllable “plant” or their combination.

### Data and statistical analyses

We used MATLAB and TISEAN (v2.1.0, TISEAN, Frankfurt, Germany) to reconstruct the attractors. We used a single factor analysis of variance (ANOVA, using MATLAB) and repeated measures (number of trials) to compare the attractor features across populations with significance set at *p* ≤ 0.05. In order to assess the healthy aging effects we compared younger adults with older adults and to assess the effect of PD, we compared healthy older adults with older adults diagnosed with PD. Finally, to assess the combined effects of aging and clinical condition, we compared younger adults with older adults diagnosed with PD.

## Results

### Reconstructed attractors

**Fig 6:**
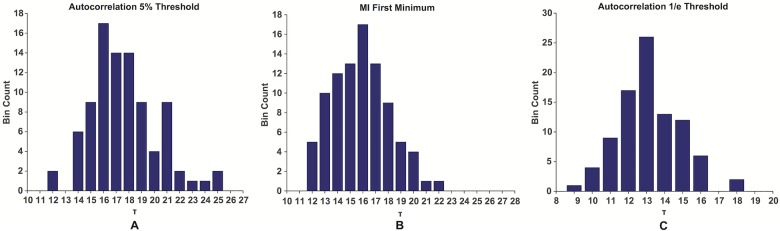
The histograms for the choice of *τ*. The histograms for all the populations obtained using the three different approaches for choosing the optimal embedding delay *τ*. The histogram for the 1/*e* threshold of the autocorrelation function (A), for the first minimum of the mutual information (B) and for the 5% threshold of the autocorrelation function (C).

We computed the optimal embedding delay *τ* and embedding dimension *m* for all ninety hold phases. We found conservative values to be *τ* = 14 and *m* = 3. Fig 6 shows the histograms obtained for the choice of the optimal embedding delay *τ*.

**Fig 7:**
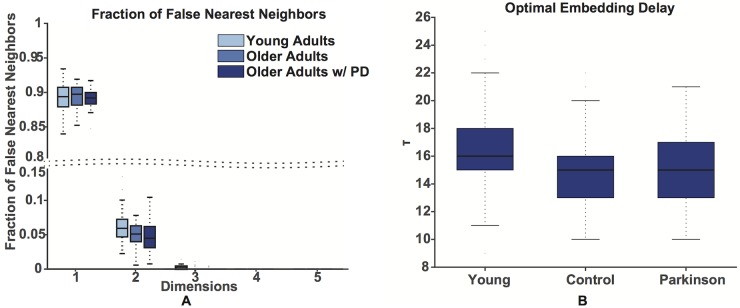
The choice of the optimal embedding dimension and delay. The fraction of the false nearest for all the three populations as a function of the optimal embedding dimension (A). The number of false nearest neighbors drops below 1% for all the three populations when *m* is chosen greater or to three. The comparison between the values of *τ* obtained with all the three approaches (1/*e* and 5% thresholds of the autocorrelation function and first minimum of the mutual information) for the three populations (B). Median, first and third quartiles are shown, whiskers show the 1.4 interquartile range values.

Fig 7B shows the comparison between the resulting *τ* values among the three populations. Fig 7A shows the fraction of false nearest neighbors for the three populations, as a function of the embedding dimension. For *m* > 3 the number of false nearest neighbors drops below 1% for all the populations.

**Fig 8:**
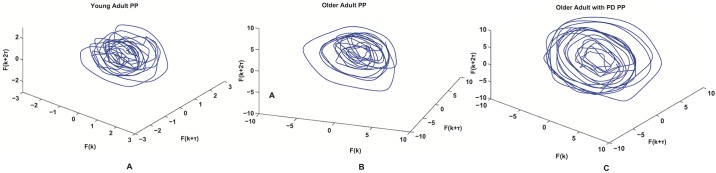
The reconstructed attractors. Reconstructed Phase Portrait (PP) for a young adult (A), an older adult (B) and an older adult with PD (C).

Fig 8 shows the reconstructed attractors for three representative subjects each one belonging to a different population. In all we analyzed 90 hold phases (3 × 30) by plotting the compression force recorded at the index finger at each time point, *F*_*k*_, against two of its delayed versions, *F*_(*k*+*τ*)_ and *F*_(*k*+2*τ*)_. That is, a 3-D plot, or an embedding dimension, *m*_0_, of three.

### Attractor features

**Fig 9:**
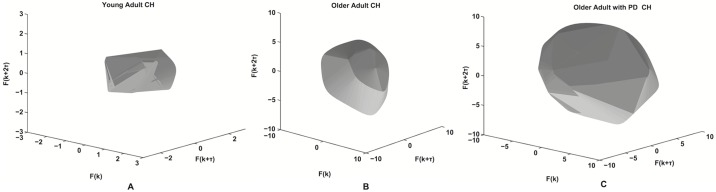
The minimal convex hulls. Minimal Convex Hulls embedding the reconstructed attractors for a young adult (left), an older adult (center) and an older adult with PD (right).

**Fig 10:**
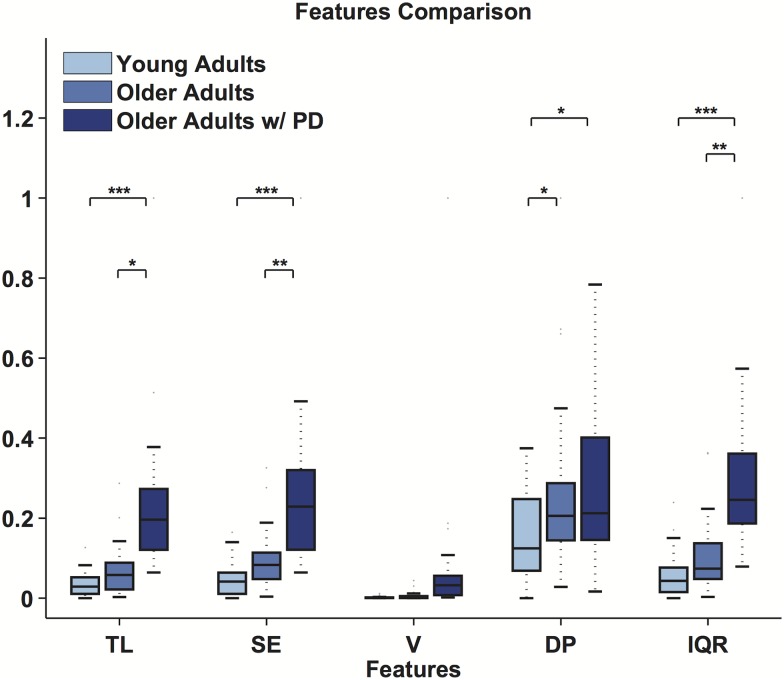
Normalized features. The comparison of the normalized features for the three populations is shown at the bottom. Statistical significance (*p* < 0.05) is indicated with an *. Median, first and third quartiles are shown, whiskers show the 1.4 interquartile range values.

The features detailed in the Methods are computed from the reconstructed phase portraits. Fig 9 shows the minimal convex hull encasing the phase portrait for a representative participant from each population. The results of the statistical analyses are reported in Table 1. We report significant effects when comparing healthy older adults and older adults affected by PD in TL (*p* = 0.0046), SE (*p* = 0.0035), and IQR (*p* = 0.0013). Only DP (*p* = 0.028) showed a statistically significant difference when comparing young healthy adults with older healthy adults. When considering the comparison between young healthy adults and older adults affected by PD, we found statistically significant effects for TL (*p* < 0.001), SE (*p* < 0.001), DP (*p* = 0.047), and IQR (*p* < 0.001). The comparisons between the geometric features for all groups are shown in Fig 10. For graphical reasons every feature was normalized among groups to lay in the [0, 1] range.

**Table 1:** ANOVA results among groups. * indicates significance of 0.05, ** indicates significance of 0.01, and *** indicates significance of 0.001.

Features	Age	Neurologic condition	Age/Neurologic condition
*TL*	*p* = 0.085	*p* = 0.0046*	*p* < 0.001***
*SE*	*p* = 0.083	*p* = 0.0035**	*p* < 0.001***
*V*	*p* = 0.19	*p* = 0.063	*p* = 0.051
*DP*	*p* = 0.028*	*p* = 0.87	*p* = 0.047*
*IQR*	*p* = 0.13	*p* = 0.0013**	*p* < 0.001***

Statistical analysis of the spatial features compared among groups. Trajectory Length (TL), Sum of the Length of the Edges of the minimal convex hull (SE), Volume of the minimal convex hull (V), Number of Distant Points (DP), and Interquartile Range of the Euclidean Distances from the centroid (IQR).

### Ellipsoid fitting

**Fig 11:**
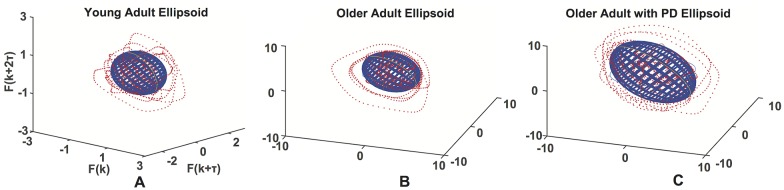
The fitted ellipsoids. Ellipsoids fitted to reconstructed attractors (red), for a young adult (A), an older adult (B) and an older adult with PD (C).

**Fig 12:**
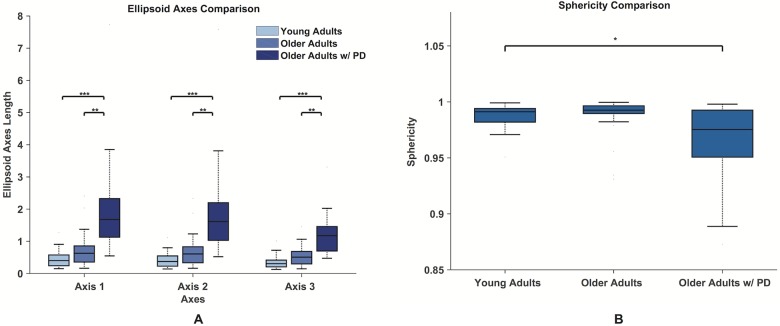
Ellipsoids axes and sphericity comparisons. Comparison between the axes of the ellipsoids fitted to the phase space points (A). Median, first and third quartiles are shown, whiskers show the 1.4 interquartile range values. The ellipsoid of the healthy young and older adult populations are more spherical and significantly smaller than the ellipsoid fitted to the minimal convex hulls of participants diagnosed with PD. The comparison of the sphericity for the three populations (B). Statistical significance (*p* < 0.05) is indicated with an *.

Representative ellipsoids of each population are shown in Fig 11. The comparison among the ellipsoids axes for each population is shown in Fig 12A. Comparing healthy young adults with healthy older adults we found only near significant differences in the principal axes of the ellipsoids (*p* = 0.062), the effects of PD were found to significantly affect the dimensions of the all three principal axes of the ellipsoids compared to healthy older and younger adults, respectively (first axis: *p* = 0.006 and *p* < 0.001; second axis: *p* = 0.006 and *p* < 0.001; third axis: *p* = 0.001 and *p* < 0.001).

The comparison among the sphericities for all three populations is shown in Fig 12B. While we do not report statistically significant age effects (*p* = 0.988) or PD effects (*p* = 0.062) on the sphericity of the phase portraits, we find a significant interaction between age and PD (*p* = 0.045).

### Phase portrait point variability

**Fig 13:**
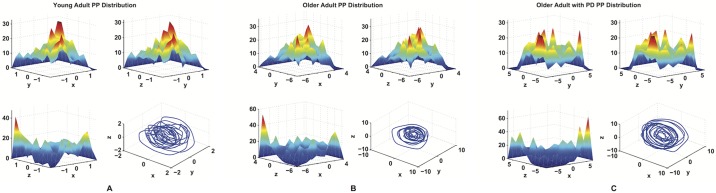
Examples of phase space points distributions. Examples of the discrete probability functions for the phase portrait of a young adult (A), an older adult (B) and an older adult with PD (C).

Since we found significant differences in the IQR value, which is related to the shape of the points distribution, we performed a qualitative analysis inspecting visually the spatial distribution of the phase space points. Fig 13 shows a comparison of the spatial distributions of points of representative phase portraits from all three populations with histograms of density of points (i.e., probability distributions) for each projection of the phase portraits. The discrete probability mass of the attractor can be estimated using histograms. The space is divided into bins and the occurrence count of points in every bin, divided by the total number of points, provides an estimation of the posterior probability in each bin. Non-uniformly spaced bins are recommended when the distributions of points in the reconstructed phase portraits are non-uniform [35]. The application of uniform bins would lead to an occurrence count that depends on how the phase trajectory crosses the intercepts of the bin. This issue is eliminated using non-uniform bins, where each dimension is divided into ten partitions such that each partition contains a fixed number of points. This requires a two-step process. First, a set of intercepts is computed along each dimension such that the dimension the histogram formed by the intercepts is uniform. Second, the higher-dimensional bins are formed as hypercubes whose boundaries are formed by the intercepts determined in the first step. The procedure is repeated for the all two-dimensional planes (namely *X* − *Y*, *Y* − *Z* and *Z* − *X*).

## Discussion

We show that the moment-to-moment dynamics of fingertip forces during spring compression at the edge of instability reveals differences in the latent attractors. This suggests the ability to detect differences in the underlying neural control strategies—and not just differences in force or force variability—across age and health conditions. This goes beyond prior work focused on the statistical and spectral properties of force variability at the edge of instability [4, 5, 8]. These new findings have clinical impact because they may provide a simple means to quantify changes in the nature sensorimotor control for dexterous manipulation with age and the progression of PD. As such they could be used as a quantitative outcome measure for disease presence, progression and treatment in PD and other neurodegenerative conditions.

There are, of course, limitations to the use of attractor reconstruction for biological systems, but we feel that those do not challenge the central finding that there were distinctly different effects of aging and PD on the neural control of dexterous manipulation. Methodologically, attractor reconstruction is an appropriate approach because we have reported that the time histories of force during spring compression at the edge of instability reveal features of a bifurcating nonlinear dynamical system [2]. So long as we assume that differences in performance across subject conditions (young vs. elderly vs. elderly with PD) were not large enough to change *τ* (the embedding delay) or *m*_0_ (the embedding dimension), then we are reasonably able to compare attractors among them. This assumption is supported by the fact that our separate estimate of these two parameters in each population did not differ.

### Attractor reconstructions as a means to characterize the neuromuscular control of manipulation

The central tenet of this work is that the phase portrait trajectories of fingertip forces—as quantified by their reconstructed attractors—are informative of neuromuscular control to stabilize the unstable spring-hand system. As discussed in, for example [36, 37], there are strong relations among the various notions of attractors and stability for dynamical systems. Therefore, detecting an attractor in the phase portrait of our manipulation task at the edge of instability—as our results show—is indicative of a stabilizing action by the neuro-musculo-skeletal system. We have published neurophysiological and brain-imaging evidence that the stabilizing control action we see during the compression of slender springs prone to buckling has a neural component [2, 5, 8, 9, 38, 39]—in addition to some contributions by muscle properties [40]. But our quantification of the dynamics of fingertip forces at the edge of instability has only gone as far as reporting that the stability of the attractor resembles that of a subcritical pitchfork bifurcation [12]. Those multiple prior findings motivated this work, where we now find that both aging and PD affect the nature of the attractor, and therefore the underlying neural control strategies for dexterous manipulation.

### Effects of healthy aging

As shown in Table 1, we find that the phase portraits of older adults demonstrate a loosening or weakening of the strength of the attractor (and thus suggest a degenerative trend in neural control strategy) compared to their younger counterparts. First, the number of distant points, DP, is significantly greater in older adults (*p* = 0.028), which hints at a weaker capability of the control action to reach the attractor (Table 1, Fig 10). A weaker control action will produce trajectories that cover a larger subset of the state space. This trend is also present in the other geometric features considered in this analysis, albeit not to a statistically significant level. A point of clarification is needed when interpreting these results as evidence of a “weaker” control action in older adults. One can also argue that a phase portrait that covers a larger subset of the phase space could be considered a stronger attractor because it is able to pull in points that are further from its center of attraction. It is here where the concept of density of the phase portrait can become critical. At first approximation, a denser phase portrait can be interpreted as a stronger control because the probability that the trajectories remain close to the attractor is higher. We therefore compared the distribution of points in the phase portraits of healthy younger and older adults, and found greater kurtosis (i.e., peakedness of the distribution, or a stronger mode) in the younger adults as shown in Fig 13. The larger phase portraits in the elderly are also more scattered and show more variability in their point distribution. Thus, this is an indicator of a weakening of their capability to drive the system state toward the attractors fixed point, despite the fact that sometimes larger phase portraits can represent stronger attractors.

In fact, these results are in agreement with prior work demonstrating age-related impairment in dynamic compensatory tracking tasks [41, 42], which takes the form of reduced efficiency of corrective motion without an increase in response latency [43]. In [43], we concluded that the age-related deficits in dynamic compensatory tasks are a functional adaptation to increased endogenous noise. The reduced efficiency of corrective actions and increased stochasticity we see here are consistent with the response of a controller subjected to increased endogenous noise in healthy aging—and likely exacerbated by the presence of PD (see below).

### Effects of neurological condition

Table 1 reports strong differences between older adults with and without PD. We readily acknowledge that it is not necessarily surprising that older adults with PD, by virtue of having a neurological disability known to degrade manipulation, tend to have larger (i.e., weaker) phase portraits compared to their healthy older counterparts, and that both of them are larger than young adults as measured by the volume and sum of edge lengths of the convex hulls. What is interesting, however, is that when compared to healthy age-matched older adults, we find significant differences in the TL and IQR features of the phase portraits in older adults with PD. This allows us to go beyond prior work [5] and disambiguate between the effects of age and PD during manipulation at the edge of instability *at the level of the nature and structure of the neural controller*.

Let us consider the differences between older adults with and without PD in the detail that our methodology allows. We find statistically significant differences in three features (Table 1, Fig 10, bottom): TL (*p* = 0.0046), SE (*p* = 0.0035), and IQR (*p* = 0.0013). As mentioned above, a significant difference in the TL feature, in particular, highlights a more chaotic behavior when PD is present, due to the increase in the stochastic component introduced by the disruption of neural control. Furthermore, the significant statistical differences in SE and IQR and the higher residuals for the ellipsoid fit (Fig 10, top right) speak to the more distributed and scattered nature of the phase portraits of the participants with PD. These differences are further illustrated in the comparison of the representative distributions of points (Fig 13), which provides information about the irregularity in the points distribution. The higher density of points around the centroid of the attractor demonstrated by the healthy subjects (both young and older adults) indicates less variability in the point distribution, compared to participants with PD who demonstrate lower densities near the attractor center.

In terms of the shape of the fitted ellipsoid (Fig 12), we find that healthy individuals, both young and older, exhibit almost spherical shapes that we attribute to the presence of a stronger attractor, with most trajectories residing inside the sphere. Their attractor is, in fact, capable of attracting points belonging to a space that is more spherical, thus its comprising points belong to a relatively symmetric 3-D space. This property is partially lost in older adults with PD, likely as a result of the neurological condition (Fig 12). The more chaotic behavior of the phase portraits coupled with the presence of a weaker attractor can be interpreted as reduced controllability during low force dexterous tasks due to the disruption of neural control associated with PD. Interestingly, this is in opposition to published results during static force exertion tasks, where the healthy individuals seem to display more variability than those with PD [20–22]. This may speak to the fact that our dynamic force production tasks at the edge of instability are more representative of ADLs than static tasks.

### Clinical implications and further developments

This work presents the application of attractor reconstruction as a tool to assess the differences in neural control during a *dynamic manipulation* task at low force magnitudes. As such, this study fills the gap with previous works [20–22] featuring analysis during *static force production*. We strongly believe in the importance of emphasizing dynamic manipulation because it represents a better approximation of ADLs, thus it gives a better insight for the treatment and the assessment of clinical conditions, such as PD.

This method successfully disambiguates the differences on the attractors of the biological dynamical system in young adults, older adults, and older adults with PD, highlighting the individual effects of aging and neurological condition (PD). The effect of healthy aging is that of a mild weakening of the attractor/control action, introducing more variability in the distribution of the points in the phase portrait. However, the neurological condition (PD) reveals itself as a partial loss of the full three-dimensional volume of the attractor, as a symptom of a weaker capability of the control action to drive points in the phase space towards the attractor itself. Both aging and PD contribute to an increasing level of chaos, suggesting that, from a nonlinear dynamic system viewpoint, an increasing level of variability in the forces is a symptom of weaker neural control.

The study of the dynamical stabilization of objects during the manipulation has pointed to activity of frontal-parietal-cerebellar networks. Those studies indicate that increasing the dexterity requirements of the task is associated with selective increases in the activity of the basal ganglia, which are active during the sustained spring compression of the SD test [39]. This study now shows that PD is a condition that changes the nature of the neural control strategy for dexterous manipulation. Therefore, further work is necessary to establish the specific mechanisms by which the nature and structure of neural control changes with the neurological deficits found in PD. However, we can speculate that our findings are compatible with other studies [44], where it is hypothesized that the PD-related motor deficit stems from a reduction of the tonic levels of dopamine in midbrain neurons. This reduction results in dysfunctional basal ganglia-thalamocortical circuits, characterized by abnormal neuronal firing patterns and pathologically synchronized oscillatory activity [45]. Such pathologically enhanced synchronization has been hypothesized to“lock-in” the motor systems, with the effect of preventing an appropriate recruitment of motor neurons for actions that are voluntary [46, 47]. The particular spatial distribution of the attractor points for subjects diagnosed with PD highlights deficits in their neural control action to efficiently drive the system state into the phase space region near the attractor fixed point, but rather to keep the system dynamics in larger limit cycles. Thus our study presents a means to deepen our understanding of the neural control of dexterous function, presenting a method that can be generalized to leg function in PD to provide a dynamical-systems characterization of the known deficits in neural control of dynamic foot-ground interactions [8]. This relatively small study represents a proof-of-principle, which now motivates and justifies the effort needed to conduct a larger study to actually determine the actual physiological mechanisms and how they are tied to the proposed measurements. A series of studies will thus follow in which advanced neurophysiological recordings will be integrated in the presented methodology to help us understand what happens at a neural level during the task execution.

**Fig 14:**
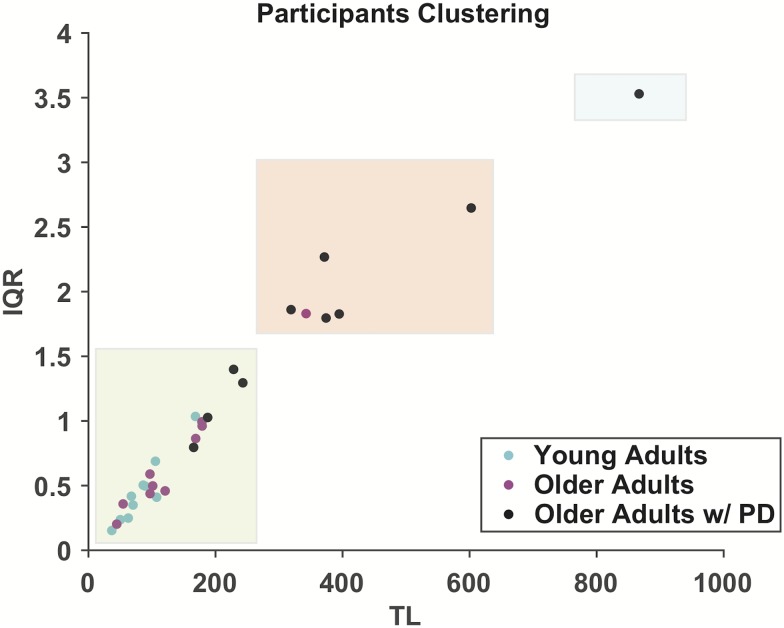
Automatic clustering of participants. The data points from every participants, averaging the three trials, were clustered using the k-means approach. The considered variables were TL and IQR. The optimal number of clusters was chosen to be three using the jump method. Healthy subjects were clustered in the first clustered, despite the age. Subjects affected by PD were mainly clustered in the second and third clusters.

There is a growing demand to understand the contribution of the natural aging process to the onset, severity, and progression of PD. In fact, several characteristics of PD coincide with those also linked to aging (i.e., changes in synapse and mitochondrial structure) [48], making the clinical assessment of the neurological condition a challenging task [49]. We believe that our results here, and others over the lifespan [4, 8, 40], open important opportunities to disambiguate the effects of natural aging from those of particular pathologies, as well as exploring sex-related differences and developing rehabilitation and treatment paradigms. Our results offer in facts an easy and portable way that can be used also to qualitatively assess the onset of clinical conditions. As an example, we used k-means clustering on the averaged TL and IQR data from all participants and chose the optimal number of clusters, in this case three, using the jump method. Fig 14 shows the results of the cluster analysis. Healthy young and older adults are grouped in in the first (green) cluster and participants with PD were primarily grouped in the second (red) cluster, although one was grouped in the third (blue) cluster. Future studies will explore is the relationship between the severity of the PD symptoms according to the Unified Parkinson’s Disease Rating Scale (UPDRS) score and the features of the reconstructed attractors.

In summary, in this study we investigated the dynamics seen while subjects use fingertip forces of low magnitudes to compress a compliant and slender spring at the edge of instability. In particular, we explored whether these dynamics differ among healthy young adults, healthy older adults and older adults diagnosed and being treated for PD. We find distinct effects of aging and PD on the dynamics at the edge of instability. Importantly, these nonlinear methods are designed to characterize the latent dynamical “attractor”, and therefore our results strongly suggest differences in the capabilities of the underlying neuromuscular controller. Given that attractor reconstruction techniques infer properties of the underlying neuromuscular controller, this work goes beyond prior reports of statistical differences in the mean, variance and frequency content of stabilizing fingertip forces across these populations [5, 8]. Therefore, this work motivates and justifies future research aimed at identifying the neurophysiological mechanisms responsible for differences in the neuromuscular control of dynamic manipulation associated with aging and PD.

## Supporting information

S1 File Dataset. CSV file with the best three hold phases for every participant. Every column is a hold phase, while the first row specifies the subject number, group and hold phase number in the following format *Group_SubjectID_HoldPhase*.
(CSV)

S2 File Code. Zip file containing the code to reproduce the results of our study.
(ZIP)
